# Not all bad decisions are alike: approach and avoidant bad decisions are associated with distinct network organization

**DOI:** 10.3389/fnins.2023.1249008

**Published:** 2023-10-09

**Authors:** Siraj Lyons, Brendan Eliot Depue

**Affiliations:** ^1^Neuroimaging Laboratory of Cognitive, Affective, and Motoric Processes, Department of Psychological and Brain Sciences, University of Louisville, Louisville, KY, United States; ^2^Department of Anatomical Sciences and Neurobiology, University of Louisville, Louisville, KY, United States

**Keywords:** decision making, Iowa gambling task, reward, personality, functional connectivity

## Abstract

**Introduction:**

Decisions under ambiguity occurs daily for everyone. Subsequently, we all deliberate upon options to initiate an action most appropriate for current goal demands. Researchers has attempted to identify factors which contribute to risk taking, alongside the neurocircuitry underpinning it. Empirically, uncertain decision making is frequently assessed using the Iowa Gambling Task (IGT). Research have reliably identified varying regions implicating two broader circuits known as the reward and salience networks. However, considerable work has focused on contrasting “good” versus “bad” decisions.

**Methods:**

The present investigation attempted a unique approach to analyzing the modified IGT acquired during fMRI (*n* = 24) and focused on active and passive bad decisions to identify potential internetwork connectivity, dissociable connectivity patterns between approach and avoidant bad decisions, and their relationship with personality traits, which can be linked with behavioral approach styles.

**Results:**

Network cluster analyses revealed general internetwork connectivity when passing (avoiding) good decks; however, the OFC was functionally disconnected from the rest of the selected brain regions when playing (approaching) bad decks. Decreased reward responsiveness was linked to increased functional connectivity between the lateral OFC and aSMG, while drive was associated with increased functional connectivity between dACC and aINS.

**Discussion:**

We report evidence that approach and avoidant bad decisions are associated with distinct neural communication patterns. Avoidant decisions were marked by substantial network integration and coherence, contrasted with the general scarcity of internetwork communication observed for approach decisions. Furthermore, the present investigation observed preliminary evidence of personality traits linked with neural communication between salience and reward evaluative networks.

## 1. Introduction

We encounter ambiguous situations which require concrete decisions on a daily basis. Empirically, decision making under uncertainty has commonly been assessed using the Iowa Gambling Task (IGT; Bechara et al., 1994). Participants select a card from one of four simultaneously presented decks with the objective of gaining as much money as possible. Two of the four decks are suboptimal, wherein the participant will lose money over time while the other two decks are associated with a net gain. The nature of each deck is unknown at the start of the task, leaving the participant to learn good from bad through the process of trial and error. In their seminal paper, Bechara et al. (1994) observed that individuals with a lesioned ventromedial PFC (vmPFC), also referred to as the medial orbitofrontal cortex (OFC), selected more cards from bad decks compared to participants without a neurological lesion. It was theorized that the vmPFC subsequently contributes to learning under ambiguous contexts. Research in the brain sciences since the first publication of the IGT has moved beyond the study of discrete regional nodes of function and toward a wider picture of neural mechanisms that contribute to risky decisions. We propose a preliminary global network organization that subserves valuation of potential reward within context-dependent goal updating throw two constituent components, namely the frontostriatal and salience networks. These networks appear to emerge from interactions of discrete regions identified within the extant literature (reviewed below) concerning uncertain or “risky” decision making.

Learning under risk requires constant, moment-to-moment evaluation of sensory information and updating strategies when behaviors are no longer goal relevant or subserve goal attainment. The OFC is situated atop an apex of a sensory hierarchy, as seen by structural and functional connectivity (Small et al., 2007). The lateral OFC shares rich connectivity with several regions responsible for sensory stimuli such as the visual cortex, through the inferior fronto-occipital fasciculus (Conner et al., 2018), and amygdala through the uncinate fasciculus (Von Der Heide et al., 2013). Facilitating higher level processing, the OFC produces associate pairs between sensory stimuli (Sadacca et al., 2018). These associations are thus represented online to construct general rules for behavioral responding. In tasks like the IGT, association pairs between decks and outcome, alongside their visual cues and physiological outcomes, facilitates learning and subsequent behavioral flexibility (Nogueira et al., 2017). As such, the lateral OFC (including the frontal operculum) occupies the role of maintaining cognitive, affective, and sensory information online (Hooker and Knight, 2006). Therefore, it is not surprising to see the importance of the OFC on IGT performance, as reported initially by Bechara et al. (1994) and more recently Ouerchefani et al. (2019); Zha et al. (2022).

The robust connections between the OFC and sensory-related areas opens the possibility that deficits in rule learning observed after lesioning could manifest through a breakdown in stimulus evaluation fed forward through the hierarchy. Sensory information from the early visual cortex is projected to anterior and ventral regions contributing to initial processing. Maximizing monetary performance taps into an expansive mesolimbic dopaminergic pathway, quintessential for reward processing, emerging from the ventral tegmental area (VTA), with efferent projections terminating in the NAcc (Ikemoto, 2007; Haber, 2014). Mesolimbic dopaminergic activity reliably increases for reward anticipation (Knutson et al., 2001), reflecting incentive, or motivational, saliency (Berridge and Robinson, 1998; Berridge, 2007; Salamone et al., 2007). Decision making in the IGT is repeatedly linked with NAcc activity (Lawrence et al., 2009; Linnet et al., 2011; Tanabe et al., 2013). Thus, NAcc activity is thought to reflect an evaluative process of stimuli value, allowing for modulation of behavioral agency in conjunction with other regions. The amygdala additionally receives dopaminergic innervation (Inglis and Moghaddam, 1999; Casey et al., 2023), and is strongly associated with the regulation of autonomic activity (Bechara et al., 1994; de Voogd et al., 2016). The dopaminergic efferent projections from the VTA to amygdala appear to be important for inducing stress (Inglis and Moghaddam, 1999) and for memories of aversive events (Frick et al., 2022). This positions the amygdala as a critical region for processing sensory information it receives to initiate shifts in physiological states and assist in encoding events which precipitated autonomic changes. Alongside value valuation from the NAcc, these processes are maintained by the OFC to guide decision making (Nogueira et al., 2017).

Additionally, for sensory information to eventually reach the OFC, attentional mechanisms must be recruited for external and internal state representation. The inferior parietal lobule (IPL) and anterior insula (aINS) are involved in the selection of behaviorally relevant stimuli passed to higher-level cortical regions. Couched between visual, auditory, and sensorimotor regions, the IPL, contributes to the integration of sensory information across multiple domains (Lynch, 1980; Zhang and Li, 2014). Damage to portions of the IPL impairs successful attentional allocation to task relevant stimuli (Shomstein et al., 2010). As such, failure to allocate attention to salient stimuli would impair paired associations maintained by the OFC. Another source of attention modulation comes from the aINS, another region of multimodal processing. The aINS works in conjunction with the IPL through various structural (Burks et al., 2017) and functional (Zhang and Li, 2014) connections for attentional modulation/allocation of multimodal representations (Weissman et al., 2006; Mason et al., 2007; Craig, 2009). Attention shifts mediated by the aINS occurs through the selection behaviorally relevant information from both internal (i.e., physiological) and external environments (Seeley et al., 2007). Furthermore, it has been suggested that the aINS contributes to gating information from consciousness (Huang et al., 2020). Thus, abnormal functioning may additionally impair rule-learning under uncertain contexts by fragmentation the process of forming paired stimulus associations.

Learning with ambiguous information requires some level of trial-and-error, where decisions impeding goal attainment ideally signal control mechanisms to update selection and outcome associations. Updating these associations requires active monitoring and regulation of top-down signals that are linked to goal maintenance, a process consistently attributed to the anterior cingulate cortex (ACC; Swick and Turken, 2002; Orr and Hester, 2012; Spunt et al., 2012; Caruana et al., 2018). While the OFC oversees relationships between stimuli and outcomes, the dACC, through its dense connections with motor regions (Paus, 2001), it thought to aide in connecting outcomes to their precipitating actions (Rudebeck et al., 2008). Therefore, the dACC potentially behaves as a mediator between cue-based associations provided by lower-order sensory information and eventual behavioral output, by signaling the need for cognitive control when requisite adjustments to goals and their maintenance is needed (Botvinick et al., 2001; Kolling et al., 2012, 2014; Shenhav et al., 2016).

Our current understanding in brain sciences suggest that brain regions do not operate as solitary entities. All regions discussed can be grouped into distinct, functional brain networks. One such network, the salience network, includes the dACC, aINS, and a subportion of the IPL known as the anterior supramarginal gyrus (aSMG). The prevailing view of the salience network includes the putative role of selecting behaviorally relevant stimuli, and subsequently influences shifts in network dynamics for further processing (Seeley et al., 2007; Sridharan et al., 2008; Menon and Uddin, 2010). The remaining structures, medial/lateral OFC and NAcc, can be organized into a frontostriatal network (Tekin and Cummings, 2002). As it is believed that both major parcellations of the OFC generally contribute to tracking ongoing rules, neural communication between it and the NAcc could underpin an evaluative process in which motivational and stimulus value is weighed against an expected outcome based on prior experiences.

Individual differences in decision making under uncertainty can be found in the extant literature focusing on personality. A predominate theory of personality was proposed by Gray, (1981, 1982), postulating two unique systems underlying behavioral approach and behavioral inhibition which is assessed using the Behavioral Approach and Inhibition scale (BAS/BIS; Carver and White, 1994). Approach- and inhibition-related traits are related to risk taking (Kim and Lee, 2011), and general performance (Franken and Muris, 2005; Suhr and Tsanadis, 2007) on gambling tasks. Carver and White (1994) noted a three-factor model constructing behavioral approach from their inventory; fun-seeking, drive, and reward responsiveness. These subconstructs are often independently associated with decision-making tasks. Franken and Muris (2005) observed a relationship between reward responsiveness and overall performance while Suhr and Tsanadis (2007) only implicated Fun Seeking. Reward responsiveness was also linked with increased anticipatory skin conduction when selecting bad decks. Neurologically, BAS fun seeking correlates with OFC activity during the IGT (Yamamoto et al., 2017). Therefore, behavioral approach is subsequently involved in reward-based decision making and psychophysiological systems, although, no clear consensus exists implicated a particular subscale with decision making.

Within the IGT literature, much of the work focuses on distinguishing neural activity associated with good vs. bad decisions. However, relatively little focus has been placed on investigating approach- or avoidant-related decisions differ neurologically. Specifically, in the IGT, there are two types of prescriptive bad decisions; playing a card from a bad deck (approach) and deciding against playing a card from a good deck (avoidant). Approach and avoidant behaviors are linked with dissociable patterns of neural activity (Davidson et al., 1990). Subsequently, we were interested in whether (1) neural activity, specifically among the frontostriatal and salience networks, differs between an approach or avoidant bad decision and (2) if activity relates to personality characteristics associated with behavioral approach. An *a priori* network for salience and frontostriatal regions were created. Specifically, the dorsal ACC, aINS, aSMG, and amygdala were selected for the salience network and the medial and lateral OFC, and NAcc were selected for the frontostriatal network. Although the canonical salience network as described by Seeley et al. (2007) did not include the amygdala, MRI investigations of risky decision making tasks have implicated the amygdala for processing sensory experiences in the IGT. Furthermore, the amygdala processes raw sensory experiences (Li et al., 1996), projects to the aINS (Mufson et al., 1981; Ghaziri et al., 2017), and may contribute to affective salience (Liberzon et al., 2003; Kong and Zweifel, 2021). It is hypothesized that approach and avoidant bad decisions will be associated with dissociable functional connectivity patterns. Additionally, general internetwork connectivity between frontostriatal and salience networks is expected. Furthermore, we expect that personality traits contributing to individual differences in approach behaviors will be associated with functional connections when considering bad decisions. We additionally expect that personality traits would be related to the speed in which an individual makes an approach or avoidant bad decision. Specifically, higher scores on the BAS subscales would be negatively correlated with approach reaction times (i.e., those more sensitive to reward will make approach decisions more quickly). Meanwhile, higher scores in BIS would positively correlate with approach reaction times, but negatively correlate with avoidant reaction times (i.e., individuals more sensitive to punishment would make approach decisions more slowly, but avoidant decisions more quickly).

## 2. Materials and methods

### 2.1. Participants

*Power considerations*—We have converging indications that our sample size (*N* = 24) was adequate. Calculating effect size using Cohen’s D for Student’s *t*-test between conditions of interest from Depue et al.’s study (*t*-test of parameter estimates/df), emotional decision making; task effect size were: Cohen’s *D* = 2.69, *r* = 0.75, medium effect size (*N* = 23). Furthermore, multiple emotional decision making studies have shown functional activation differences between the conditions of interest using 16–20 participants (Ochsner et al., 2002, 2004; Phan et al., 2005). We also seek to use individual differences in neural measures with personality variables. Our previous work with 23 participants found a relationship between personality and functional activation within *a priori* ROIs (Cohen’s *D* = 1.25, *r* = 0.54, medium effect size). Using G*Power software for *a priori* estimates using our past effect sizes (0.75, 0.54), indicates that a sample size between 15 and 30 per group will be sufficient.

Participants were recruited from an undergraduate student population through SONA, a participant and study management service. A total of 27 participants were recruited (16 females), with the average age being 22.19 (SD = 4.26). All participants were 18 years old or older. Due to MRI data quality (*n* = 1) and missing data (*n* = 2), 24 participants were statistically analyzed. The study was approved by the University of Louisville Institutional Review Board. This study was a part of a larger investigation concerned with personality and its relationship cognitive, social, and interpersonal functioning.

### 2.2. Modified Iowa gambling task

**FIGURE 1:**
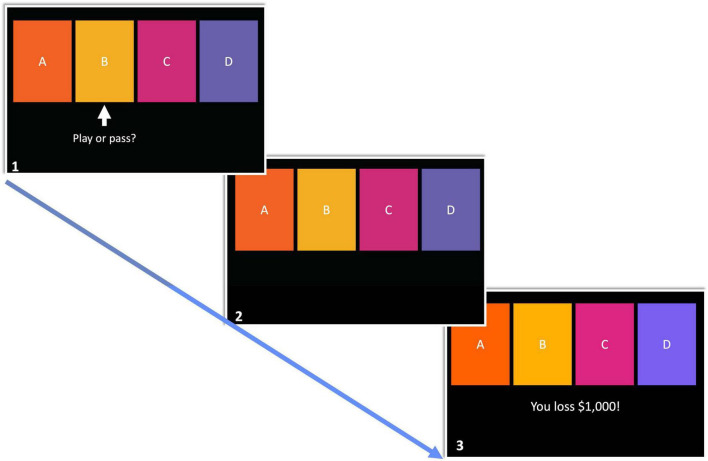
The modified Iowa Gambling Task comprised of three phases. (1) In the first phase, participants have 4,000 ms to choose from the highlighted deck. (2) The remaining time in phase 1 following decision is spent waiting until feedback is presented. (3) Absolute value of decision outcome is presented.

A modified version of the IGT (hereafter referred to as mIGT) was utilized in this investigation while participants were in the scanner. A key distinction with the mIGT pertains to trial presentation. Instead of freely selecting from four simultaneously presented decks, participants were prompted to play or pass a from one of the four decks at a time (see Figure 1, scene 1), leading participants to gain experiences with each deck. If a deck is played, feedback was presented using net-outcomes. For example, if the card played has a $250 dollar reward, but a punishment of $1,250, the net-outcome of –$1,000 was presented. The order of playable decks was pseudorandomized. Restricting decisions to an individual deck in each trial reduces variance between participants as it prevents the development of idiosyncratic strategies which may influence the effect individual differences investigated in the present study (Fogleman et al., 2017). Furthermore, presenting net-outcomes following the decision to play or pass reduces inter-participant variability in attending to feedback (e.g., strongly attending to winning feedback, but not losing feedback). The mIGT as described here has been implemented in past research (Cauffman et al., 2010; Tanabe et al., 2013; Fogleman et al., 2017). An exemplary trial flow is presented in Figure 1. Due to the construction of the mIGT, it was possible to functionally dissociate active from avoidant decision making. For the purpose of the present investigation, an approach bad decision is defined as playing from a bad deck (i.e., decks “A” and “B”) while avoidant bad decisions are defined as passing good decks (i.e., decks “C” and “D”). The mIGT was comprised of 40 trials.^1^ The task was presented via Eprime2 on a computer monitor placed at the end of the scanner bore.

### 2.3. Behavioral approach scale

To index individual differences in reward responsiveness, the Behavioral Approach Scale (BAS) was administered (Carver and White, 1994). This scale is administered alongside a set of seven questions indexing behavioral inhibition and four filler questions indexing neither approach or inhibition. The BAS factors into three subscales; Fun Seeking, Drive, and Reward Responsiveness. Previous investigations enumerated constructs unique to each subscale. Fun seeking relates to the desire for new experiences and has been associated with impulsivity. Drive indexes behavioral motivation to pursue reward. Reward responsiveness reflects positive affect following reward receipt (Carver and White, 1994).

### 2.4. Neuroimaging acquisition

#### 2.4.1. Structural MRI

Structural MRI images were acquired using a T1-weighted magnetization-prepared rapid gradient-echo sequence (MPRAGE) in 208 sagittal slices with these following parameters: TE = 2.26 ms, TR = 1700 ms, flip angle = 9°, FoV = 204 mm, and voxel size = 0.8 mm × 0.8 mm × 0.8 mm. Structural MRI images were collected using a Siemens 3T Skyra MR scanner using a 20-channel head coil for radiofrequency reception. To reduce movement artifacts, foam padding was placed within the head coil. Participants wore earplugs to reduce scanner noise and received instructions through headphones worn during scanning.

#### 2.4.2. Functional MRI

Blood oxygenation level-dependent (BOLD) scans were acquired using gradient-echo T2*-weighted echoplanar imaging using the following parameters: (TE = 28 ms, TR = 2,000 ms, flip angle = 9.0 degrees, FoV = 204 mm, voxel size = 2 mm × 2 mm × 2 mm, 78 interleaved slices). Slices were oriented obliquely along the AC-PC line. A total of 166 scans were collected during the mIGT. The same procedures for controlling movement and presenting instructions were used during the functional scan.

### 2.5. Procedures

Informed consent was acquired from participants prior to data collection. The investigation comprised of two laboratory sessions. First, the mIGT was completed while participants were at the MRI center. The total MRI session lasted upward of 1.5 h. Afterward, questionnaire data was acquired at the main laboratory location. Upon satisfying the study requirements, participants were compensated with $50.

### 2.6. Imaging data analysis

All analyses for the present investigation were conducted in CONN, a functional connectivity toolbox for MATLAB 2022a (CONN version 20.b; Whitfield-Gabrieli and Nieto-Castanon, 2012) dependent on SPM12 (Penny et al., 2007). Functional MRI images were coregistered to their respective structural image and centered. Data underwent slice time correction to correct temporal drift which occurs during functional acquisition. Outliers in BOLD data and excessive movement were investigated using default settings (Intermediate 97th percentile), which identifies voxels with BOLD signal exceeding a z-score of 5 and movement beyond 0.9 mm. No participants were found to be outliers or moved excessively during scanning. Functional and structural images were then normalized to standardized MNI space and segmented into gray matter, white matter, and CSF classes according to default tissue probability maps using SPM12 unified segmentation and normalization procedure. Functional images were smoothed at 5 mm kernel using a Gaussian filter. Following preprocessing, functional images were then denoised using a 0.01—infinity Hz filter and underwent linear detrending. Denoised and detrended functional data were entered in a first-level linear regression and Pearson *r* correlations were calculated across *a priori* ROIs which comprise the salience and reward networks. Correlations were Fischer’s *z*-score transformed in the second-level regression. Reward responsiveness and drive subscale scores were mean centered and entered as a covariate within the second-level regression. Alpha criterion was set to a False Discovery Rate (FDR) corrected *p*-value of 0.05 at cluster level.

Functional network connectivity was assessed using parametric multivariate statistics, the default statistical pipeline for network clustering in CONN (Jafri et al., 2008). ROIs from our defined salience and reward networks were entered and were grouped using hierarchical clustering procedures that consider anatomical distance and functional activity similarity. We selected this data driven approach to observe whether our *a priori* network behaves uniquely between approach and avoidant bad decisions. Functional connections were evaluated in a pairwise fashion within and between hierarchically defined clusters. This procedure was followed for both bad play and good pass conditions. Connection threshold was set at an uncorrected *p* < 0.05 and cluster corrected to *p*_FDR_ < 0.05. Next, BAS subscales and BIS data were regressed onto seed-based functional connectivity analyses to assess unique relationships with personality characteristics.

ROIs were selected from the default network and anatomical atlases, which were derived from an independent component analysis of fMRI scans from the human connectome project and both Harvard-Oxford and Automated Anatomical atlases, respectively. These atlases are default in CONN. As neither atlas contains the medial and lateral OFC, the second version of the Automated Anatomical Atlas (AAL2) was imported into CONN (Rolls et al., 2015).

### 2.7. Behavioral data analyses

To investigate differences in reaction time, a paired-sample *t*-test were completed. Reaction times (RTs) for plays from decks A and B were averaged to produce a single “approach bad decision” value, while RTs for passes from decks C and D were averaged to produce a single “avoidant bad decision” value (see Table 1). Relationships between drive and RTs for good passes and bad plays were assessed using Pearson’s *r* correlations. Spearman’s ρ was calculated to assess the relationship between reward responsiveness and reaction times as it was not normally distributed. Behavioral data were analyzed in R (version 4.2.3).

**TABLE 1:** 

Decision type	RT	SD	Min	Max
Approach bad	1,534.22	766.15	436	3,852
Avoidant bad	1,277.75	654.99	398	3,689

“Approach bad” refers to playing from decks A or B. “Avoidant bad” refers to passing decks C or D.

## 3. Results

### 3.1. Behavioral results

Reaction times for approach were significantly longer than avoidant decisions, *t*(23) = 3.11, *p* = 0.005. Furthermore, participants additionally made more avoidant decisions than approach, *t*(23) = −5.69, *p* < 0.001. Descriptive statistics are presented in Table 1. Neither approach or avoidant RTs and card selections were related to behavioral approach subscales, *p*s > 0.477. Behavioral inhibition was negatively correlated with total selections made from bad decks, *r* = −0.522, *p* = 0.009. However, when controlling for multiple tests (*Holm correction*) the relationship was no longer significant, *p* = 0.116.

### 3.2. Network cluster analysis

#### 3.2.1. Passing from good decks

**FIGURE 2:**
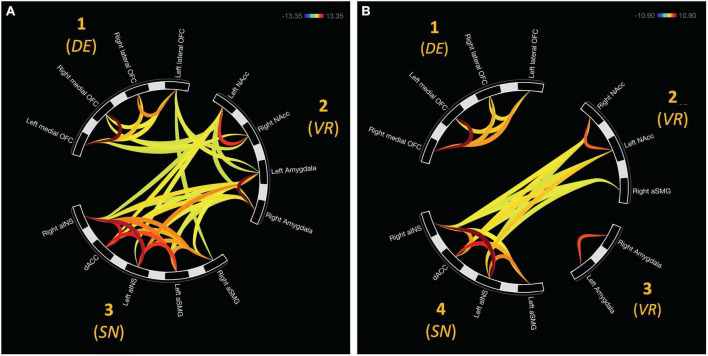
Functional network clusters associated with bad decisions in the mIGT. **(A)** ROIs were hierachically organized into three distinct clusters when making avoidant bad decisions. All cluster were functionally connected. **(B)** Approach bad decisions displayed unique clustering relative to avoidant decisions. Internetwork connectivity was restricted to salience (aINS, dACC, and left aSMG) and value representation (NAcc and right aSMG). DE, decision expectancy; VR, value representation; SN, salience network.

For avoidant bad decisions, ROIs were grouped into three distinct clusters. Cluster one was comprised of the bilateral medial OFC and lateral OFC, *F*_(2, 22)_ = 90.94, *p*_FDR_ < 0.00001. Cluster two consisted of the bilateral NAcc and amygdala, *F*_(2, 22)_ = 79.71, *p*_FDR_ < 0.00001. Bilateral aINS, aSMG, and dACC were found in cluster three, *F*_(2, 22)_ = 73.33, *p*_FDR_ < 0.00001. These clusters are depicted in Figure 2A.

**FIGURE 3:**
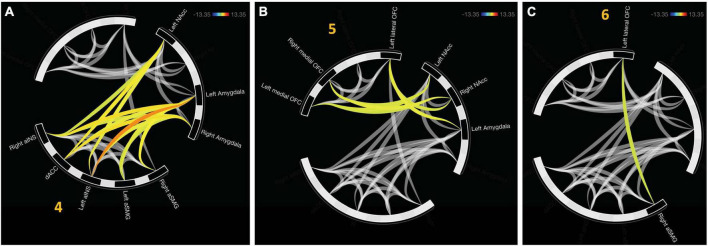
Functional connectivity emerged between hierarchically defined clusters. **(A)** Cluster 4 represents functional communication between regions representative of the salience network (aINS, dACC, and SMG) and value representation (NAcc and Amygdala). **(B)** Cluster 5 displays connectivity between online maintenance (medial and lateral OFC) and value representation. **(C)** In the final cluster, communication between salience (right aSMG) and online maintenance (left lateral OFC).

There were three additional clusters representing functional connectivity between other clusters. Cluster four was the first between-network result spanning between clusters two and three, *F*_(2, 22)_ = 16.94, *p*_FDR_ < 0.0001. Cluster five representing internetwork connectivity between cluster one and two, *F*_(2, 22)_ = 10.42, *p*_FDR_ < 0.001. Cluster six connects clusters three and one, *F*_(2, 22)_ = 4.21, *p*_FDR_ < 0.05. Internetwork connectivity is presented in Figure 3.

#### 3.2.2. Playing from bad decks

Network organization was assessed using hierarchical clustering analyses which. Four distinct clusters emerged from these analyses for approach bad decisions. Cluster one consisted of the bilateral aINS, aSMG, and dACC, *F*_(2, 20)_ = 44.04, *p*_FDR_ < 0.00001. Cluster two consisted of the bilateral medial and lateral OFC, *F*_(2, 20)_ = 35.57, *p*_FDR_ < 0.0001. Cluster three was represented by the bilateral amygdala, *F*_(1, 21)_ = 43.92, *p*_FDR_ < 0.0001. Cluster four consisted of the bilateral NAcc and right aSMG, *F*_(2, 20)_ = 13.50, *p*_FDR_ < 0.001. These clusters are depicted in Figure 2B.

**FIGURE 4:**
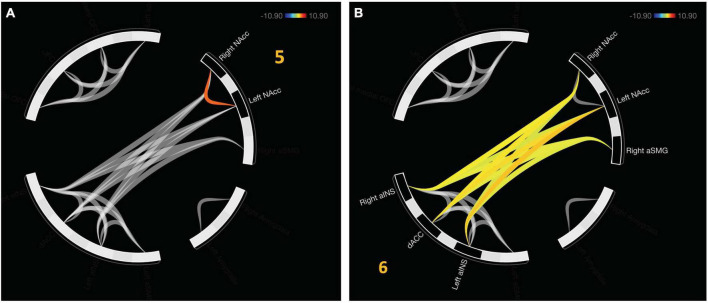
Approach bad decision had intercluster communication, albeit less profound relative to avoidant decisions. **(A)** Cluster 5 indicates unique functional connectivity between left and right NAcc distinct from its broader cluster encompassing the bilateral NAcc and right aSMG. **(B)** Cluster 6 represents communication between the salience network (aINS and dACC) with value representation.

An additional cluster was identified which represents functional connectivity between networks. Cluster 5 represents a distinct, functional connectivity between the left and right NAcc, *F*_(2,20)_ = 13.5, *p*_FDR_ < 0.001. Cluster 6 displayed connectivity between clusters one and four, *F*_(2, 20)_ = 16.54, *p*_FDR_ < 0.001. A detailed depiction of intra- and internetwork connectivity is depicted in Figure 4.

### 3.3. Personality traits and specific regional connectivity

Univariate, seed-based ROI analyses were conducted to reveal unique connectivity patterns to drive and reward responsiveness subscales of the behavioral approach scale. Reward responsiveness, when accounting for drive, was positively predictive of increased negative functional connectivity between the left aSMG and lateral OFC, *t*(19) = −3.30, *p*_FDR_ = 0.046 when passing from good decks. No significant results were found for playing bad decks, *p*s_FDR_ > 0.28.

**FIGURE 5:**
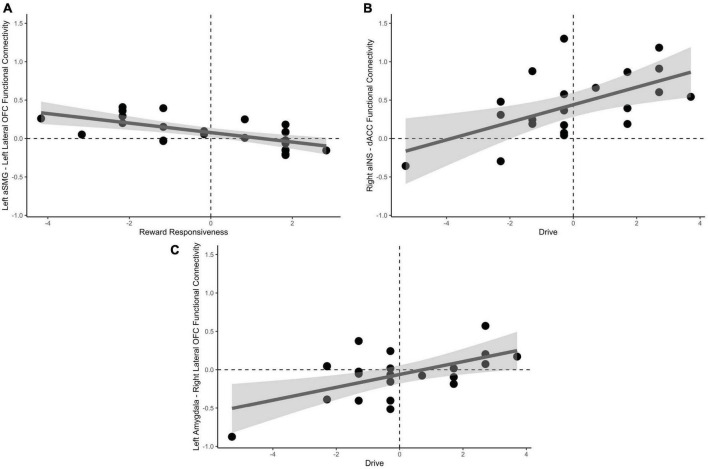
Relationships between behavioral approach measures and functional connectivity when considering risky choices. **(A)** Decreased reward responsiveness significantly related to increased functional connectivity between the left aSMG and lateral OFC. **(B)** Greater endorsement of behavioral drive was associated with increased functional connectivity between the right aINS and the dACC. **(C)** Left amygdala and right lateral OFC functional connectivity was associated with greater behavioral drive when approaching bad decision. aSMG, anterior supramarginal gyrus; OFC, orbitofrontal cortex; aINS, anterior insula; dACC, dorsal anterior cingulate cortex.

Drive, when accounting for reward responsiveness, was positively predictive of increased positive functional connectivity between the dACC and right aINS, *t*(19) = 3.59, *p*_FDR_ = 0.023, in addition to increased positive functional connectivity between right lateral OFC and left amygdala, *t*(19) = 3.34, *p*_FDR_ = 0.034 when approaching bad decisions. No significant results were found for avoidant bad decisions *ps*_FDR_ > 0.23. These results are depicted in Figure 5.

No associations were observed with the fun seeking or behavioral inhibition scales.

## 4. Discussion

The purpose of this investigation was to examine functional connectivity that distinguishes differing forms of bad decisions. While a considerable amount of literature investigating the IGT focuses on good vs. bad decks, bad decisions can be made by playing from a bad deck or passing the opportunity to play from a good deck. Generally, this can be regarded as approach and avoidant decisions, respectively. It is known that approach and avoidance/withdrawal is associated with distinct neural patterns (Davidson et al., 1990). Furthermore, it is known that personality traits, such as the tendency to engage in approach- or avoidant- related behaviors indexed by the BAS/BIS, is related to decision-making in risky contexts. Thus, these data aimed to add to the IGT, and more generally risk taking, literature by presenting evidencing reporting the specific neural relationship with approach and avoidance, suboptimal decisions, and their potential link with personality traits.

### 4.1. Behavior

We hypothesized that BAS subscales would negatively correlate with approach bad decisions while BIS would positively correlate with approach RTs and negatively correlate with avoidant RTs. However, we observed that reaction times were not related to personality characteristics involved in reward seeking as indexed by the BAS/BIS (Carver and White, 1994). Although, we note that, from behavioral data that participants were quicker to make avoidant decisions in the mIGT, thus the sample exhibited a general aversion toward a “riskier” version of a bad decision. These results replicate data presented in Cauffman et al. (2010) were young adults (i.e., individuals in their early 20 s) predominately made more avoidant than approach related decision.

### 4.2. Global network organization

We hypothesized, broadly, that approach and avoidant bad decisions will be associated with distinct global network organization, as revealed by hierarchical cluster analyses. Furthermore, we expected the aINS, dACC, and aSMG to comprise the SN, medial and lateral OFC and NAcc to comprise the frontostriatal network. Overall, the present results support these hypotheses with a few notable exceptions listed below (see Figures 2A, B). Generally, regions were organized in a more coherent global network in avoidant, relative to approach, decisions. ROIs were organized by a hierarchical clustering procedure indicating, three constituent networks involved in both avoiding and approaching bad decisions. First, inclusion of medial and lateral OFC represents a *decision expectancy network* where new information (i.e., the current trial presented) is compared against recent outcomes and general task rules, which are held online. The amygdala and NAcc together formed a *value representation network* where time-sensitive stimuli were evaluated. Lastly, the bilateral aINS, dACC, and aSMG formed the canonical salience network, involved in detecting and monitoring salient cues in the internal and external environment. However, when approaching bad decisions, global network organization appeared more fragmented (Figure 2B). We observed that the bilateral amygdala and NAcc no longer formed the *value representation network*, as they individually formed separate groupings. Additionally, the *decision expectancy network* (medial and lateral OFC) was functionally disconnected from the rest of the global network. Thus, when approaching bad decisions, only the salience and NAcc-based *value representation* networks were integrated, as compared to full network integration during avoiding bad decisions.

#### 4.2.1. Value representation network

Our hypotheses combined the amygdala with the salience network due to evidence linking it with sensory processing and affective salience. However, we observed that the amygdala may actually contribute to value representation in conjunction with the NAcc when considering choices under risk. Connections between the amygdala and NAcc are well documented. The amygdala may casually modulate excitability of the NAcc to promote reward-seeking behavior (Ambroggi et al., 2008; Stuber et al., 2011). Damage to the basolateral nucleus impairs decision making in the rodent version of the IGT by enhancing risk-seeking behavior (Zeeb and Winstanley, 2011). Interrupting neural communication between these structures could thereby disrupt neural representation of value. Because the OFC maintains task characteristics (Nogueira et al., 2017) and its stimulus paired associates (Sadacca et al., 2018), information generated by dysfunctional value representation promotes associations counterproductive for goal attainment. In approach bad decisions, a lack of functional connectivity was observed between the amygdala and NAcc (Figure 2B), therefore no longer being functionally organized in the network comprising value representation. This interpretation is nuanced by our observed functional connectivity between the right amygdala and left NAcc for avoidant bad decisions (Figure 2A), as such avoidant behavior may be protective against potential, perceived harmful outcomes. Thus, functional connectivity between these regions could reflect devaluation, perhaps lead by the amygdala as dopaminergic activity within the structure is tied with stress and aversion (Inglis and Moghaddam, 1999; Frick et al., 2022). Despite an interesting possibility, the nature of the present investigation cannot speak to the specific directionality between amygdala and NAcc communication.

#### 4.2.2. Decision expectancy network

Hierarchical clustering results show that the bilateral OFC, both medial and lateral parcellations, are functionally disconnected from the rest of the *a priori* network for approach compared to avoidant bad decisions (i.e., playing from bad decks vs. passing good decks). The literature shows strong structural connectivity between the OFC and regions responsible for processing sensory and affective information (Hooker and Knight, 2006). Furthermore, within the context of decision-making, it is thought that the OFC, particularly its lateral subdivision, is responsible for maintaining recent outcomes for consideration while individuals evaluate current task demands (Nogueira et al., 2017). Meanwhile, medial portions of the OFC have largely been attributed to representing value of choices and hedonic experiences. Our results suggest value and outcome representation may not be fully neurologically communicated when individuals make an approach bad decision. Fiuzat et al. (2017) observed deficits in adaptive behavior following value devaluation in rhesus monkeys following cross disconnection of the OFC and amygdala. As the fragmented value representation network (NAcc) and the salience network were functionally connected, both were not connected to the decision expectancy network, implicating a lack of top-down rule guidance, which may explain approach bad decisions.

#### 4.2.3. Saliency network

Approach and avoidant bad decisions were both linked to functional connectivity between the value representation and salience networks. The putative role of the salience network involves the selection of behaviorally relevant stimuli for successful goal attainment (Seeley et al., 2007). Activity within the salience network has been identified as a potential contributor to IGT performance (Lawrence et al., 2008; Li et al., 2010). Our results provide evidence that differing forms of bad decisions appear to utilize similar connectivity patterns between saliency and value representation. However, during approach, the canonical salience network identified for avoidant bad decisions lacked the right aSMG, as it was grouped with the NAcc. Hierarchical clustering considers the similarity in functional activity over a time series and regional distance in its clustering procedures (Jafri et al., 2008). Because the right aSMG was organized alongside the salience network in avoidant decisions, this would point to this region engaging a functionally separate role upon approach. Admittedly, the reason behind this is not intuitively apparent. However, research indicates that the broader IPL, including the aSMG, engages in multimodal representation which assists in the context formation for both external environmental and internal states (Lynch, 1980; Downar et al., 2002; Desai et al., 2013), thereby contributing to evaluating value by representing the internal affective state. Alternatively, its involvement in action planning may represent the one component of behavioral, or incentive, salience for pursuing potentially valuable options.

### 4.3. Regressions with behavior

We next correlated functional connectivity of the individual ROI-to-ROI parings with subscales of the behavioral approach and inhibition scales (BAS/BIS) during the decision phase of the mIGT. Results indicate that decreased Reward Responsiveness subscale of the BAS was correlated with increased functional connectivity between the left aSMG and lateral OFC. This pathway was previously indicated as being important for avoidant responses, perhaps by increased updating of external context and internal states (represented in the aSMG) being communicated to the decision expectancy in the OFC. The Drive subscale of the BAS, an index of behavioral motivation to seek reward, was related to increased functional connectivity between dACC and aINS, in addition to the right lateral OFC and left amygdala when approaching bad decisions. As previously stated, the dACC in connection with the aINS may subserve the processes of recruiting attentional mechanisms (Botvinick et al., 2001; Shenhav et al., 2016) and monitoring behavioral consequences, to flexibly modulate goal orientation (Kolling et al., 2012, 2014). This process would necessarily be greater during approach, than avoidant decisions. We additionally observed a positive relationship with Drive and functional connectivity between the left amygdala and right lateral OFC when approaching bad decisions. The lateral OFC and amygdala may become more functionally connected as value representation is communicated within the hierarchy (Rolls et al., 2015), which likely increases with increased Drive personality traits and makes approach decisions more attractive as a mechanism of enhanced risk seeking or tolerance (Jung et al., 2018).

### 4.4. Limitations and future directions

Several limitations are present. Participants, on average, selected cards less than 2 s after presentation, causing the neural activity assessed during the selection period to include both pre-decision and anticipatory phases. It is thought that neural mechanisms underlying these stages are functionally distinct (Knutson et al., 2001). Therefore, further work is required to (1) increase confidence that the current investigation did not return spurious findings and (2) extend upon the present findings by completing more nuanced analyses to better understand the interaction between trait Reward Responsiveness and Drive, their neurobiological foundations, and evaluating risk. Furthermore, a larger sample size can address other limitations such as an inequal distribution between males and females present in the current dataset. Furthermore, the IGT literature suggests sex differences wherein females make more selections from disadvantageous decks (e.g., A and B) relative to males (van den Bos et al., 2013). In fact, Bolla et al. (2004) reported activity in the OFC, a region of interest in the present investigation, was different between males and females during the task, potentially representing a neurophysiological basis of performance. This proposition is further supported by other investigations reporting sex differences in the OFC more generally (Domes et al., 2010; McGlade et al., 2015). As such, it is warranted to focus on potential sex differences between approach and avoidant bad decisions. Lastly, bad decisions on the IGT have been described as ambiguous to the amount of conscious processing involved and therefore, prescriptive in nature as defined by the researchers (Wang et al., 2012). It would be of interest to compare these results with an investigation wherein subjective reports of decision type are considered.

## 5. Conclusion

Behaviorally, we observed a general bias toward avoidant bad decisions across the general sample. Furthermore, approach and avoidant bad decisions are characterized by distinct global network organization wherein avoidant decisions were characterized by strong internetwork communication, contrasted to the functional disconnection of decision expectancy and fragmentation of the value representation constituent networks. Therefore, it is possible that network organization, may reflect stimuli (e.g., physiological arousal, visual cues, and affective states) integration under risky decision making. Avoidant decisions, despite rich internetwork connectivity, may occur following value devaluation, perhaps through functionally connectivity between the NAcc and amygdala. Approach bad decisions instead occurs following sparse internetwork connectivity absent of an effective application of task rules accumulated through updating paired stimulus associates onto perceived value of approaching, which is additionally altered as the amygdala is not integrated into the value representation network. Trait behavioral approach was implicated with functional connectivity in both avoidant and approach decisions. Lower responsiveness to reward (e.g., reward responsiveness) may correspond to increased context updating through the association of external and internal states though functional connectivity between the lateral OFC and aSMG. Motivation to peruse reward (e.g., drive), is linked to both enhanced attention and monitoring of choice outcomes (aINS and dACC functional connectivity) and risk seeking (left lateral OFC and right amygdala functional connectivity). Broadly, we report preliminary evidence that avoidant and approach bad decisions are represented by unique global network organizations, with constituent functional connectivity patterns related to facets of reward seeking personality traits.

## Data availability statement

The raw data supporting the conclusions of this article will be made available by the authors, without undue reservation.

## Ethics statement

The studies involving humans were approved by the University of Louisville–Internal Review Board. The studies were conducted in accordance with the local legislation and institutional requirements. The participants provided their written informed consent to participate in this study.

## Author contributions

SL conducted formal data analysis and drafted the manuscript. Both authors conceptualized the research question investigated in the manuscript and reviewed/edited the document.

## Footnotes

1 It is more common to administer 100 trials in the mIGT. However, the nature of the larger study necessitated truncating the mIGT to 40 trials to attenuate the possible risk of participant attrition.
